# Prehospital physiological parameters related illness severity scores can accurately discriminate the severe/critical state in adult patients with COVID-19

**DOI:** 10.1080/07853890.2023.2239829

**Published:** 2023-07-25

**Authors:** Chen Li, Kaili Wang, Liang Wu, Bing Song, Junyuan Tan, Haibin Su

**Affiliations:** a Department of Hepatology, The Fifth Medical Center of Chinese PLA General Hospital, Beijing, China; b Department of Infectious Diseases, The Fifth Medical Center of Chinese PLA General Hospital, Beijing, China; c Medical Service Department, The Fifth Medical Center of Chinese PLA General Hospital, Beijing, China

**Keywords:** National early warning score 2, coronavirus disease 2019, prehospital, severity, physiological parameters

## Abstract

**Background:**

Whether the National Early Warning Score 2 (NEWS2) can effectively discriminate the severe/critical state of patients with coronavirus disease 2019 (COVID-19) at the prehospital stage remains unknown. We aimed to assess the performance of NEWS2 in rapidly discriminating severe/critical COVID-19 and its relationship with prehospital medical services.

**Methods:**

Six illness severity scores of 414 patients were calculated at the prehospital stage. Receiver operating characteristic curves were generated to explore the ability of these scores to discriminate severe/critical patients from mild/moderate patients. A logistic regression analysis was conducted to evaluate independent predictors associated with severe/critical state.

**Results:**

The age, numbers of comorbidities, prehospital care workload, consumption of medical human resources, and illness severity scores of severe/critical patients were higher than those of mild/moderate patients (*p* < 0.05). When NEWS2 scores >2, the sensitivity, specificity, positive predictive value, and negative predictive value were 93.5%, 90.7%, 74.1%, and 98.0%, respectively. The C-statistic of NEWS2 (0.963) was higher than that of quick Sequential Organ Failure Assessment (0.680, *p* < 0.001), CRB-65 (0.879, *p* < 0.001), Rapid Acute Physiology Score (0.692, *p* < 0.001), and Rapid Emergency Medicine Score (0.879, *p* < 0.001). NEWS2 was positively correlated with the numbers of prehospital treatment measures (*r* = 0.732, *p* < 0.001), numbers of medical staff (*r* = 0.615, *p* < 0.001), and total transport time (*r* = 0.595, *p* < 0.001). Age ≥65 years (OR = 5.43, *p* = 0.016), hypertension (OR = 5.39, *p* < 0.001), active malignancy (OR = 5.94, *p* = 0.005), and NEWS2 scores >2 (OR = 124.88, *p* < 0.001) were independent predictors to discriminate severe/critical patients. Oxygen saturation (SpO_2_) (OR =1.87, *p* < 0.001) was the unique independent predictor to discriminate false positive patients from true positive patients.

**Conclusions:**

Prehospital NEWS2 can accurately and rapidly discriminate severe/critical COVID-19 during the Omicron variant wave. High levels of NEWS2 indicate an increase in prehospital care workload and consumption of medical human resources.

## Introduction

Coronavirus disease 2019 (COVID-19), caused by severe acute respiratory syndrome coronavirus 2 (SARS-CoV-2), is a global public health event, which seriously threatens human health. Beijing experienced the SARS-CoV-2 Omicron variant wave from November 2022 to January 2023 [1]. The clinical process and prognosis of COVID-19 are highly variable, ranging from asymptomatic infection to severe/critical state [2]. Some severe patients may develop acute respiratory distress syndrome and multiple organ failure, even die, in a short period of time [3], whereas non-severe patients have a good prognosis. Furthermore, there may be significant differences in treatment strategies, available medical resource allocation, and consumption of medical human resources between the two types of patients. Therefore, which patients with COVID-19 are at high risk of serious state must be determined as soon as possible. Biomarkers of hematological parameters, cytokines, liver enzymes, coagulation parameters, and immune cells collected from blood and bronchoalveolar lavages have been proved to be useful for risk stratification to predict severe and fatal patients with COVID-19 [4]. The diagnostic model composed of white blood cells, CD4 positive T cells, and D-dimer can effectively discriminate the severe/critical state from the mild/moderate state in COVID-19 caused by SARS-CoV-2 Omicron variant BA.2 infection. This model shows a C-statistic of 0.962, a sensitivity of 97.14%, and a specificity of 90.27% [5]. However, such diagnostic models and biomarkers must be obtained by laboratory testing, which are inconvenient, time consuming, and uneconomical. Some studies have used the National Early Warning Score 2 (NEWS2) [6,7], Rapid Emergency Medicine Score (REMS) [8], CRB-65 [9], and other illness severity scores, which are based on rapidly obtainable physiological parameters after admission during the 2020 outbreak, to perform risk stratification, predict the prognosis, and optimize the management of patients with satisfactory results. NEWS2 is a clinical tool recommended by the World Health Organization to assess and early recognition the deteriorating patients with COVID-19. When its score is greater than 5, the intrahospital mortality of patients with COVID-19 significantly increases, with C-statistic of 0.84–0.88 [6, 7]. When REMS ≥3, the 30-day mortality rate of patients with COVID-19 is 27.9%, the C-statistic is 0.793, the sensitivity is 100%, and the specificity is 11.6% [8]. When the threshold of CRB-65 is 1, its C-statistic is 0.80, sensitivity is 0.83, and specificity is 0.69, which is similar to the efficacy of NEWS2 in predicting death of patients with COVID-19 [9]. If the patients with severe/critical COVID-19 can be easily, timely, and accurately screened using these scores in the prehospital management, reasonably arranged available medical resources, enhanced early warning, and improved prognosis may be achieved.

However, whether these physiological parameters related illness severity scores can accurately discriminate the severe/critical state at the prehospital stage has not been explored well. Moreover, whether these prehospital illness severity scores have a correlation with prehospital care workload and consumption of medical human resources is unknown. To fill this gap, this study aimed to evaluate the performance of six physiological parameters related illness severity scores that are commonly used in emergency departments in discriminating the severe/critical COVID-19 at the prehospital stage and to explore the relationship between the best performer and prehospital care workload and consumption of medical human resources. The independent predictors for discriminating severe/critical COVID-19, the differences in clinical characteristics between false positive and true positive cases of the best performer, and the independent predictors of false positive cases were also analyzed in this study.

## Materials and methods

### Patient enrollment

**Figure 1.**
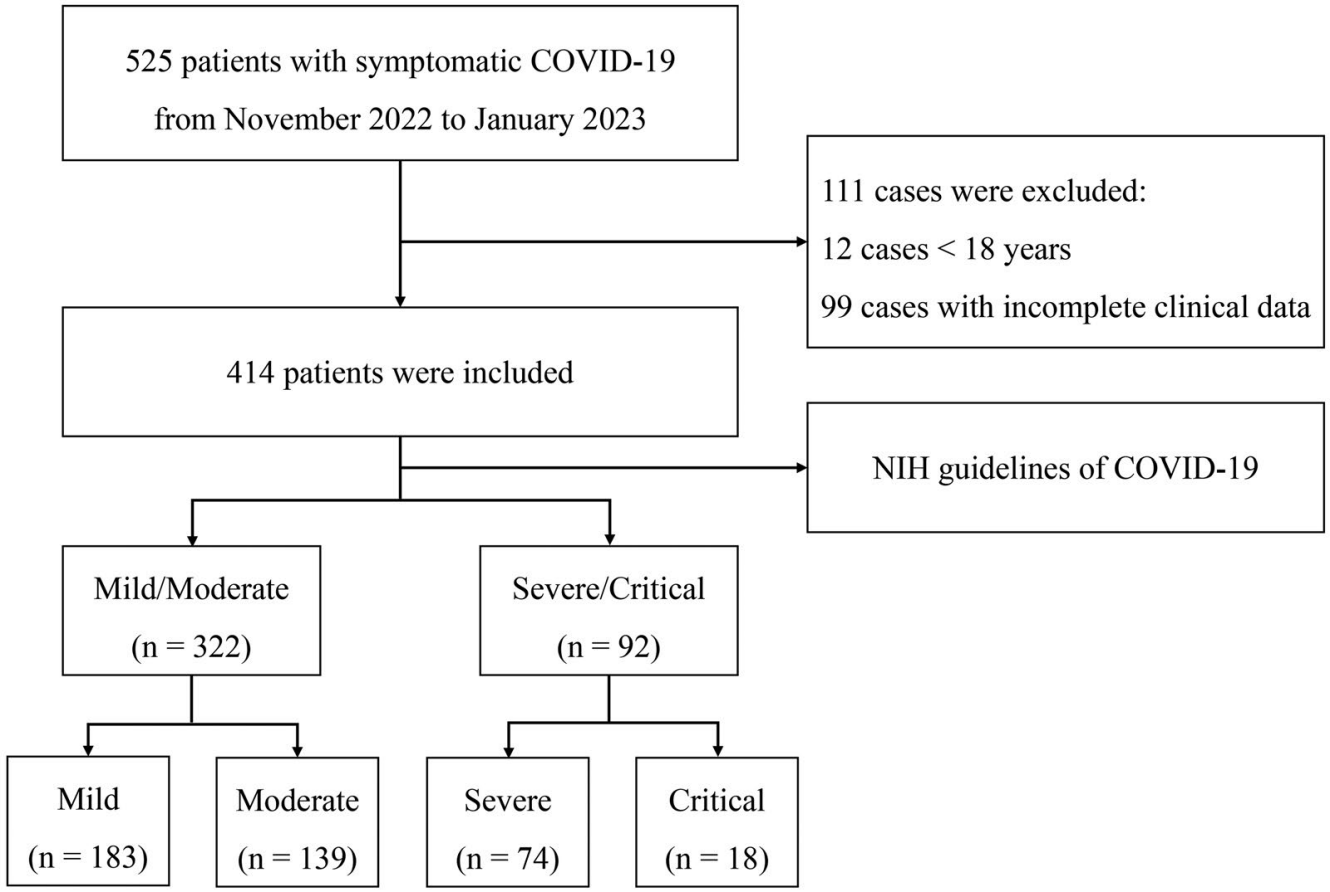
Outline of the screening and case selection protocol.

This retrospective and observational study included adult patients with COVID-19 who were transferred from the fever clinic or emergency department of other hospitals in Beijing, China to the Fifth Medical Center of Chinese PLA General Hospital which is a specialized institution for infectious diseases by negative pressure ambulance from November 2022 to January 2023. The inclusion criteria were diagnosis of COVID-19 with symptomatic infection by standard definitions. The exclusion criteria were age <18 years and incomplete clinical date. A total of 525 patients satisfied the grouping criteria. Of these patients, 111 cases were excluded, 12 cases <18 years, and 99 cases were described with incomplete clinical date. Finally, 414 patients were selected in this study (Figure 1).

### Prehospital transport setting

All patients were diagnosed with symptomatic COVID-19 by positive SARS-CoV-2 reverse transcription polymerase chain reaction tests in nasopharyngeal swab and had any of the various signs and symptoms of COVID-19. According to the illness severity, some patients received oxygen therapy, transfusion, electrocardiogram (ECG) monitoring, mechanical ventilation, and stretcher or wheelchair handling during the prehospital transport period. We collected clinical data of patients, including age, sex, comorbidities, vital signs, oxygen saturation (SpO_2_), mental status, and performance status at the beginning of prehospital transport. We also collected transport parameters of patients, including treatment measures, use of stretcher or wheelchair, numbers of medical staff, and work time during the prehospital transport period. In consideration of the different transport routes of patients, the transport time we calculated did not include the time spent on the way between the two hospitals. Total transport time for each patient = numbers of medical staff × (transport time from the first hospital to the ambulance + transport time from the ambulance to the ward in our hospital). After transportation, patients were treated in regular ward or intensive care unit (ICU) based on their conditions.

### Study design

We considered whether the patients developed serious (severe/critical) COVID-19 in 72 h after admission as the primary observation endpoint. The patients were divided into severe/critical and mild/moderate groups in accordance with the National Institutes of Health (NIH)’s guidelines for severity of illness categories of COVID-19 [2]. The scores based on quick Sequential Organ Failure Assessment (qSOFA), Rapid Acute Physiology Score (RAPS), REMS, CRB-65, NEWS2, and Pandemic Respiratory Infection Emergency System Triage (PRIEST) of all patients at the beginning of prehospital transport were calculated. Receiver operating characteristic (ROC) curves were generated to explore the ability of the six illness severity scores to discriminate severe/critical patients from mild/moderate patients. Independent predictors were analyzed to discriminate severe/critical patients from mild/moderate patients and discriminate false positive cases from true positive cases by logistic regression.

## Definitions

The severity of illness categories of COVID-19 was based on the NIH guidelines. Mild illness was defined as patients having the signs and symptoms of COVID-19 but do not have shortness of breath, dyspnea, or abnormal chest imaging. Moderate illness was defined as patients having lower respiratory disease during clinical assessment or imaging and SpO_2_ ≥94% on room air at sea level. Severe illness was defined as patients having SpO_2_ <94% on room air at sea level, a ratio of partial pressure arterial oxygen to fraction of inspired oxygen <300 mmHg, a respiratory rate >30 breaths/min, or lung infiltrates >50%. Critical illness was defined as patients having respiratory failure, septic shock, and/or multiple organ dysfunction [2].qSOFA consists of the three clinical parameters of altered mentation, respiratory rate, and systolic blood pressure (SBP), which are scored from 0 to 1, providing an overall score between 0 and 3 [10]. RAPS consists of the four clinical parameters of pulse rate, mean arterial pressure, respiratory rate, and Glasgow Coma Scale (GCS), which are scored from 0 to 4, providing an overall score range from 0 to 16 [11]. REMS consists of the four clinical parameters of RAPS, in addition to age and SpO_2_, providing a total score between 0 and 26 [12]. CRB-65 uses the four clinical parameters of confusion, respiratory rate, blood pressure, and age, each scoring 1 when positive and 0 if negative, to give an overall score between 0 and 4 [13]. NEWS2 is calculated by the seven clinical parameters of respiratory rate, SpO_2_, pulse rate, SBP, temperature, consciousness, and oxygen therapy, which are scored from 0 to 3, providing a total score between 0 and 20 [14]. PRIEST consists of the seven clinical parameters of NEWS2, in addition to age, sex, and performance status, providing an overall score between 0 and 29 [15].

### Statistical analysis

Categorical variables were expressed as percentages (%), whereas continuous variables were described as mean ± SD or median (interquartile range, Q1–Q3). Shapiro-Wilk test and quantile-quantile plot were used to assess the normality of the distribution of variables. Continuous variables were compared by conducting independent-samples t-test. The Mann–Whitney U test was performed to compare the parameters under non-normal distribution. Categorical data were compared by conducting chi-square test or continuous correction chi-square test. The correlation between the two groups of variables was calculated using the Spearman method. Trend test of categorical data was carried out using the linear-by-linear association approach. The sensitivity, specificity, positive predictive value (PPV), and negative predictive value (NPV) of the illness severity scores for discriminating serious patients were calculated by ROC curves. Youden index = sensitivity + specificity −1. The C-statistics from the two illness severity scores were compared by using the DeLong’s method. Univariate logistic regression analysis was first used to screen candidate factors. Candidate variables (*p* < 0.1) were used as inputs for multivariate logistic regression analysis following a forward stepwise approach (p-in: 0.05, p-out: 0.10). All statistical analyses were conducted using SPSS 23.0 (IBM, Armonk, NY). *p* < 0.05 was considered statistically significant in the analyses.

## Results

### Baseline clinical characteristics of patients

This study included 414 patients (183 mild cases, 139 moderate cases, 74 severe cases, and 18 critical cases) with symptomatic COVID-19 with an average age of 51.0 (33.0–84.0) years; 39.9% of them were ≥65 years, and 64.7% of them were male. Of the patients, 53.4% had comorbidities, and the common comorbidities of them were hypertension (33.6%), chronic cardiac disease (29.0%), and diabetes (16.4%). The main physiological parameters at the prehospital transport stage showed that the SpO_2_ was 97.0 (96.0–99.0) %, the temperature was 36.8 (36.4–37.3) °C, the respiratory rate was 19.0 (18.0–20.0) breaths/minute, the pulse rate was 78.0 (74.0–88.0) breaths/minute, and the SBP was 128.0 (119.8–138.0) mmHg. The main prehospital care showed that 27.5%, 12.3%, 17.9%, and 2.9% of the patients received oxygen therapy, transfusion, ECG monitoring, and mechanical ventilation, respectively. Also, 32.9% of the patients used stretchers or wheelchairs during prehospital transport. The total transport time was 8.0 (6.0–40.0) minutes. The NEWS2 and PRIEST scores of patients were 1.0 (0.0–3.0) and 3.0 (1.0–10.0), respectively. A total of 16 patients died during hospitalization, with an in-hospital mortality rate of 3.9% (Table 1).

**Table 1.** Clinical characteristics of patients with COVID-19.

Variables	All patients (*n* = 414)	Severe/critical (*n* = 92)	Mild/moderate (*n* = 322)	*P*
Age (years)	51.0 (33.0–84.0)	90.5 (80.3–95.0)	43.0 (30.0–64.3)	<0.001
≥65 years, n (%)	165 (39.9)	85 (92.4)	80 (24.8)	<0.001
Male, n (%)	268 (64.7)	66 (71.7)	202 (62.7)	0.111
Comorbidities, n (%)				
Hypertension	139 (33.6)	69 (75.0)	70 (21.7)	<0.001
Diabetes	68 (16.4)	38 (41.3)	30 (9.3)	<0.001
Chronic cardiac disease	120 (29.0)	58 (63.0)	62 (19.3)	<0.001
Chronic pulmonary disease	27 (6.5)	16 (17.4)	11 (3.4)	<0.001
Chronic urinary disease	52 (12.6)	26 (28.3)	26 (8.1)	<0.001
Cerebrovascular disease	60 (14.5)	33 (35.9)	27 (8.4)	<0.001
Chronic gastroenterology and liver disease	24 (5.8)	7 (7.6)	17 (5.3)	0.399
Active malignancy	33 (8.0)	15 (16.3)	18 (5.6)	0.001
Numbers of comorbidities	1.0 (0.0–2.0)	3.0 (2.0–4.0)	0.0 (0.0–1.0)	<0.001
Prehospital physiological parameters				
SpO_2_ (%)	97.0 (96.0–99.0)	93.0 (91.3–94.8)	98.0 (97.0–99.0)	<0.001
Temperature (°C)	36.8 (36.4–37.3)	36.9 (36.5–37.6)	36.7 (36.4–37.2)	0.036
Respiratory rate (breaths/minute)	19.0 (18.0–20.0)	20.0 (18.0–20.8)	18.0 (18.0–20.0)	<0.001
Pulse rate (beats/minute)	78.0 (74.0–88.0)	79.0 (72.0–91.0)	78.0 (74.0–86.0)	0.792
SBP (mmHg)	128.0 (119.8–138.0)	135.0 (124.3–149.8)	125.5 (119.0–135.0)	<0.001
DBP (mmHg)	78.0 (72.0–85.0)	76.5 (71.0–84.8)	78.0 (73.0–85.3)	0.091
MAP (mmHg)	94.3 (89.3–102.0)	96.0 (89.4–104.0)	94.0 (89.3–100.8)	0.236
GCS	15.0 (15.0–15.0)	15.0 (12.3–15.0)	15.0 (15.0–15.0)	<0.001
Prehospital care				
Oxygen therapy, n (%)	114 (27.5)	92 (100.0)	22 (6.8)	<0.001
Transfusion, n (%)	51 (12.3)	49 (53.3)	2 (0.6)	<0.001
ECG monitoring, n (%)	74 (17.9)	67 (72.8)	7 (2.2)	<0.001
Mechanical ventilation, n (%)	12 (2.9)	12 (13.0)	0 (0.0)	<0.001
Use stretcher or wheelchair, n (%)	136 (32.9)	81 (88.0)	55 (17.1)	<0.001
Numbers of treatment measures	0.0 (0.0–2.0)	3.0 (2.0–4.0)	0.0 (0.0–0.0)	<0.001
Number of medical staff	1.0 (1.0–2.0)	2.0 (2.0–2.0)	1.0 (1.0–1.0)	<0.001
Total transport time (minutes)	8.0 (6.0–40.0)	44.0 (42.0–46.0)	7.0 (6.0–10.0)	<0.001
Illness severity scores				
qSOFA	0.0 (0.0–0.0)	0.0 (0.0–1.0)	0.0 (0.0–0.0)	<0.001
RAPS	0.0 (0.0–2.0)	1.0 (0.0–2.0)	0.0 (0.0–0.0)	<0.001
REMS	2.0 (0.0–6.0)	7.0 (6.0–8.0)	2.0 (0.0–5.0)	<0.001
CRB-65	0.0 (0.0–1.0)	1.0 (1.0–2.0)	0.0 (0.0–1.0)	<0.001
NEWS2	1.0 (0.0–3.0)	6.0 (4.0–8.0)	0.0 (0.0–1.3)	<0.001
PRIEST	3.0 (1.0–10.0)	13.0 (11.0–16.0)	2.0 (1.0–4.0)	<0.001
In-hospital mortality rate, n (%)	16 (3.9)	16 (17.4)	0 (0.0)	<0.001

COVID-19, coronavirus disease 2019; SpO_2_, oxygen saturation; SBP, systolic blood pressure; DBP, diastolic blood pressure; MAP, mean arterial pressure; GCS, Glasgow coma scale; ECG, electrocardiogram; qSOFA, quick Sequential Organ Failure Assessment; RAPS, Rapid Acute Physiology Score; REMS, Rapid Emergency Medicine Score; NEWS2, National Early Warning Score 2; PRIEST, Pandemic Respiratory Infection Emergency System Triage.

A total of 92 (22.2%) patients were diagnosed with severe/critical COVID-19. The age, proportion of ≥65 years, proportion of comorbidities (except for chronic gastroenterology and liver disease), numbers of comorbidities, temperature, respiratory rate, SBP, prehospital care workload, number of medical staff, total transport time, six illness severity scores, and in-hospital mortality rate of the severe/critical group were higher than those of the mild/moderate group (*p* < 0.05). By contrast, the SpO_2_ and GCS scores of the severe/critical group were lower than those of the mild/moderate group (*p* < 0.05) (Table 1).

### Performance of the six illness severity scores for discriminating the severe/critical state

**Figure 2.**
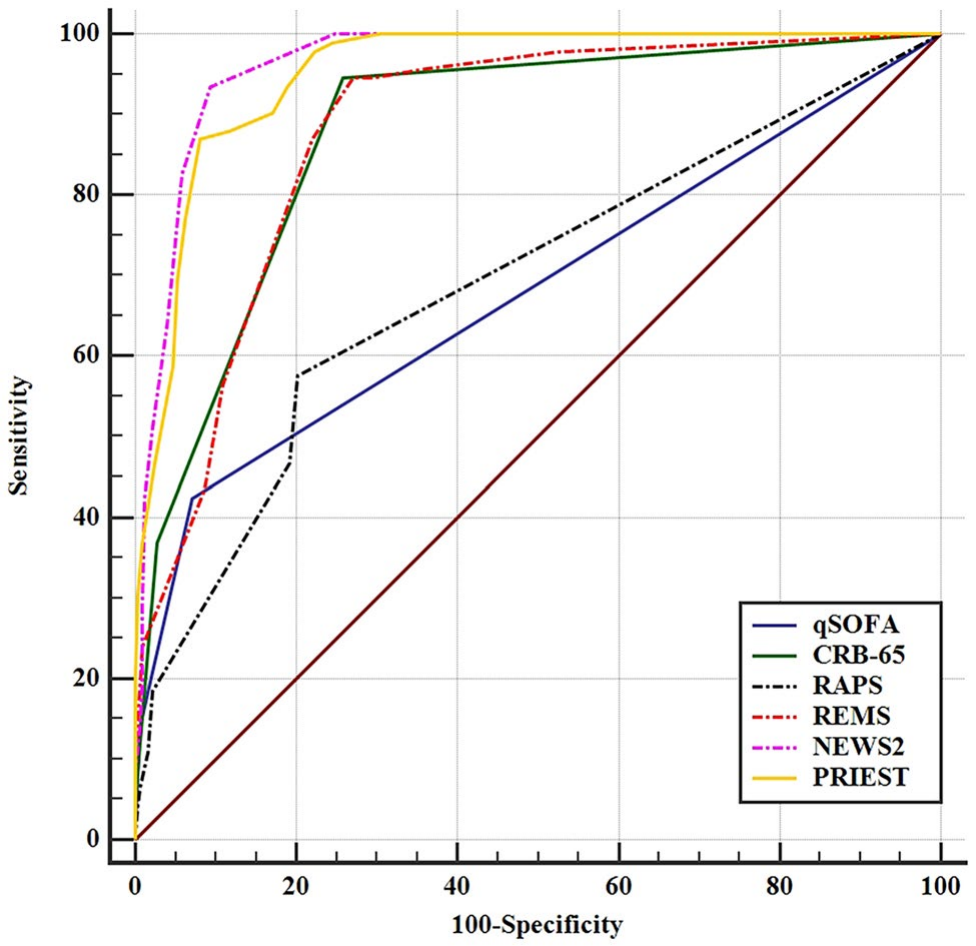
The receiver operating characteristic curves of the six illness severity scores for discriminate the severe/critical state.

The C-statistic, threshold, Youden index, sensitivity, specificity, PPV, and NPV of NEWS2 scores were 0.963 (95% CI, 0.940–0.979), >2, 0.842, 93.5 (95% CI, 86.3–97.6) %, 90.7 (95% CI, 87.0–93.6) %, 74.1 (95% CI, 65.2–81.8) %, and 98.0 (95% CI, 95.7–99.3) % by ROC curves, respectively. The C-statistics of NEWS2 scores (0.963) and PRIEST scores (0.952) were much higher than those of qSOFA scores (0.680, *p* < 0.001), CRB-65 scores (0.879, *p* < 0.001), RAPS scores (0.692, *p* < 0.001), and REMS scores (0.879, *p* < 0.001) by DeLong’s method. The C-statistic of NEWS2 scores was slightly higher than that of PRIEST scores, although the difference was not statistically significant (*p* = 0.101). However, the Youden index and sensitivity of NEWS2 scores were better than those of PRIEST scores (Figure 2; Table 2).

**Table 2.** Performance of the six illness severity scores in predicting the severe/critical state.

Illness severity scores	C-statistic (95% CI)	Threshold	Youden indices	Sensitivity (%) (95% CI)	Specificity (%) (95% CI)	PPV (%) (95% CI)	NPV (%) (95% CI)	*P_1_*	*P_2_*
qSOFA	0.680(0.633–0.725)	>0	0.353	42.4(32.1–53.1)	92.9(89.5–95.4)	62.9(49.7–74.8)	84.9(80.8–88.5)	<0.001	<0.001
CRB-65	0.879(0.844–0.909)	>0	0.688	94.6(87.8–98.2)	74.2(69.1–78.9)	51.2(43.4–58.9)	98.0(95.3–99.3)	<0.001	<0.001
RAPS	0.692(0.645–0.736)	>0	0.374	57.6(46.9–67.9)	79.8(75.0–84.1)	44.9(35.7–54.3)	86.8(82.4–90.5)	<0.001	<0.001
REMS	0.879(0.843–0.909)	>4	0.678	94.6(87.8–98.2)	73.0(67.8–77.8)	50.0(42.3–57.7)	97.9(95.2–99.3)	<0.001	<0.001
NEWS2	0.963(0.940–0.979)	>2	0.842	93.5(86.3–97.6)	90.7(87.0–93.6)	74.1(65.2–81.8)	98.0(95.7–99.3)	/	0.101
PRIEST	0.952(0.927–0.971)	>9	0.789	87.0(78.3–93.1)	91.9(88.4–94.7)	75.5(66.2–83.3)	96.1(93.3–98.0)	0.101	/

PPV, positive predictive value; NPV, negative predictive value; qSOFA, quick Sequential Organ Failure Assessment; RAPS, Rapid Acute Physiology Score; REMS, Rapid Emergency Medicine Score; NEWS2, National Early Warning Score 2; PRIEST, Pandemic Respiratory Infection Emergency System Triage.

*P_1_*: Comparison of other illness severity scores with NEWS2 scores; *P_2_*: Comparison of other illness severity scores with PRIEST scores.

### Relationship between NEWS2 scores, the severity of COVID-19, and the in-hospital outcomes

**Figure 3.**
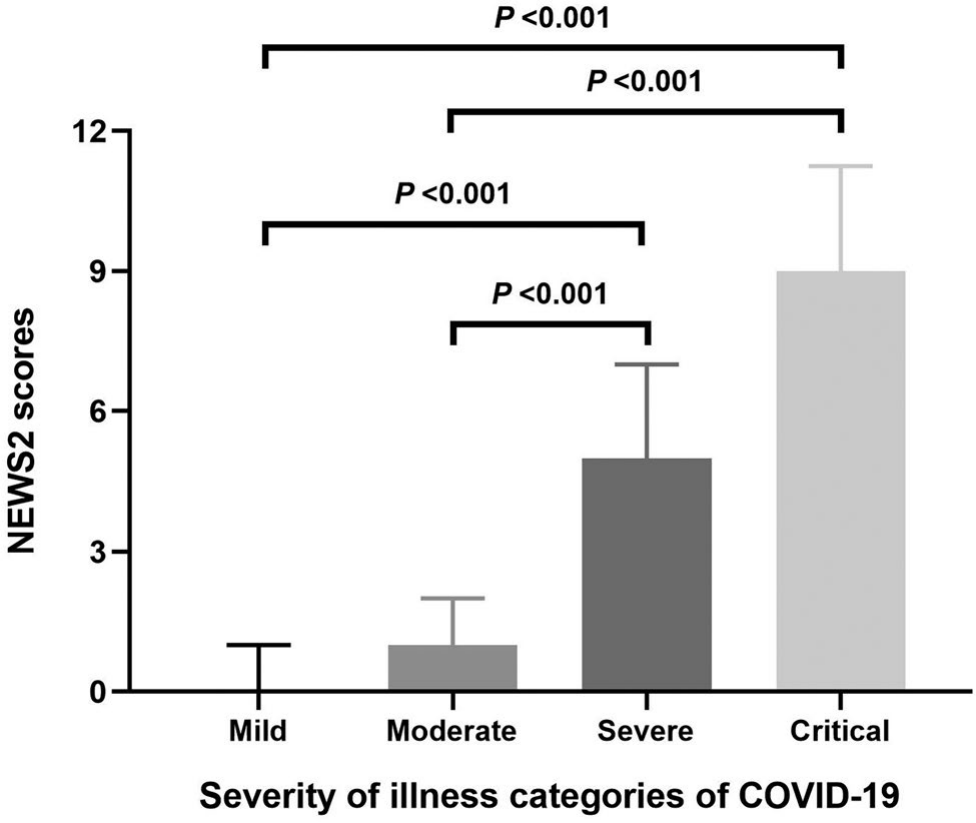
The NEWS2 scores of the four illness categories of patients with COVID-19.

The NEWS2 scores of mild, moderate, severe, and critical in patients with COVID-19 were 0.0 (0.0–1.0), 1.0 (0.0–2.0), 5.0 (4.0–7.0), and 9.0 (5.0–11.3), respectively. There was an overall difference in the NEWS2 scores of the four illness categories of patients (*p* < 0.001). The NEWS2 scores of severe and critical patients were all higher than those of mild (*p* < 0.001) and moderate (*p* < 0.001) patients (Figure 3).

**Figure 4.**
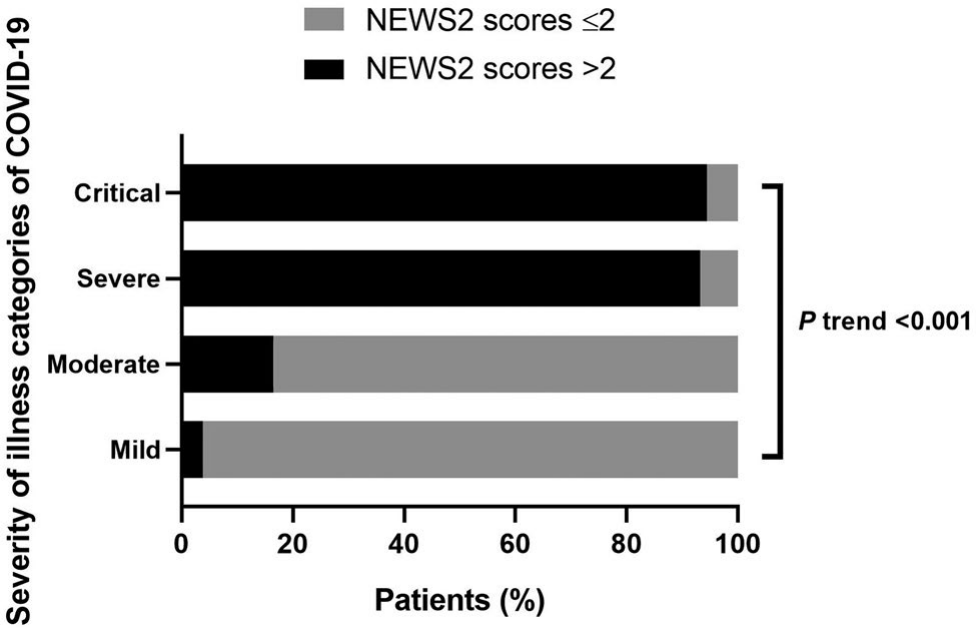
The proportion of NEWS2 scores >2 of the four illness categories of patients.

The proportion of the NEWS2 scores >2 of mild, moderate, severe, and critical in patients with COVID-19 was 3.8% (7/183), 16.5% (23/139), 93.2% (69/74), and 94.4% (17/18), respectively. There was an overall difference in the proportion of NEWS2 scores >2 of the four illness categories of patients (*p* < 0.001). The proportion of NEWS2 scores >2 in the four groups also showed a linear increasing trend with the severity of COVID-19 (*P* trend <0.001) (Figure 4).

The NEWS2 scores of non-survivor patients (7.5(5.0–11.0)) were higher than those of survivor patients (1.0 (0.0–3.0), *p* < 0.001). The in-hospital mortality rate of patients with NEWS2 scores >2 (13.8%) was higher than those of patients with NEWS2 scores ≤2 (0.0%, *p* < 0.001).

### Relationship between NEWS2 scores and age, number of comorbidities, and prehospital care workload

The NEWS2 scores of patients were positively correlated with age (*r* = 0.478, *p* < 0.001), numbers of comorbidities (*r* = 0.542, *p* < 0.001), numbers of treatment measures (*r* = 0.732, *p* < 0.001), number of medical staff (*r* = 0.615, *p* < 0.001), and total transport time (*r* = 0.595, *p* < 0.001) by Spearman method. The age, numbers of comorbidities, numbers of treatment measures, number of medical staff, and total transport time of patients with NEWS2 scores >2 were all higher than those of patients with NEWS2 scores ≤2 (*p* < 0.001) (Table 3).

**Table 3.** Comparisons of age, numbers of comorbidities and prehospital care workload between NEWS2 score >2 and NEWS2 score ≤2.

Variables	NEWS2 scores >2 (*n* = 116)	NEWS2 scores ≤2 (*n* = 298)	*P*
Age (years)	89.0 (77.3–94.0)	42.0 (29.0–60.0)	<0.001
Numbers of comorbidities	3.0 (2.0–4.0)	0.0 (0.0–1.0)	<0.001
Numbers of treatment measures	3.0 (2.0–4.0)	0.0 (0.0–0.0)	<0.001
Number of medical staff	2.0 (2.0–2.0)	1.0 (1.0–1.0)	<0.001
Total transport time (minutes)	44.0 (38.0–46.0)	7.0 (6.0–9.0)	<0.001

NEWS2, National Early Warning Score 2.

### Independent predictors for discriminating the severe/critical state

**Figure 5.**
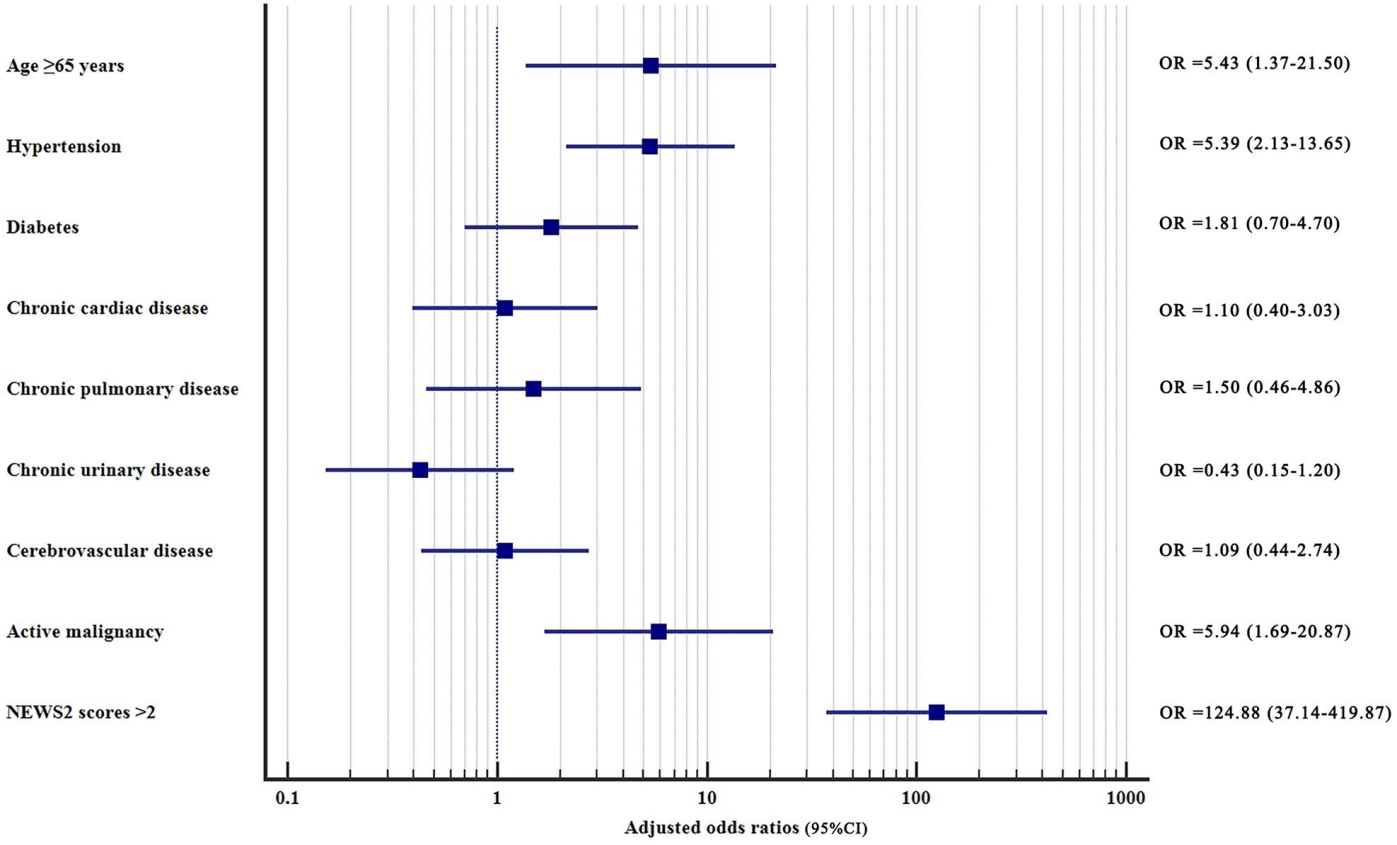
Multivariate logistic regression of independent factors predicting the severe/critical state in patients with COVID-19.

The patients who developed severe or critical COVID-19 were used as the dependent variable, and age ≥65 years, male, comorbidities, and NEWS2 scores >2 were used as the independent variables in the univariate logistic regression. Age ≥65 years, hypertension, diabetes, chronic cardiac disease, chronic pulmonary disease, chronic urinary disease, cerebrovascular disease, active malignancy, and NEWS2 scores >2 were screened out as meaningful variables and used as inputs for multivariate logistic regression (*p* < 0.1) (Table 4). Multivariate logistic regression showed that age ≥65 years (adjusted OR =5.43, *p* = 0.016), hypertension (adjusted OR =5.39, *p* < 0.001), active malignancy (adjusted OR =5.94, *p* = 0.005), and NEWS2 scores >2 (adjusted OR =124.88, *p* < 0.001) were independent predictors for severe/critical categories in patients with COVID-19 (Table 4) (Figure 5).

**Table 4.** Independent predictors for severe/critical categories in patients with COVID-19.

Variables	Univariate logistic regression		Multivariate logistic regression	
	OR (95% CI)	*P*	Adjusted OR (95% CI)	*P*
Age ≥65 years	36.73(16.32–82.66)	<0.001	5.43(1.37–21.50)	0.016
Male	1.51(0.91–2.50)	0.112		
Hypertension	10.80(6.29–18.55)	<0.001	5.39(2.13–13.65)	<0.001
Diabetes	6.85(3.91–11.99)	<0.001	1.81(0.70–4.70)	0.221
Chronic cardiac disease	7.15(4.31–11.86)	<0.001	1.10(0.40–3.03)	0.862
Chronic pulmonary disease	5.95(2.65–13.35)	<0.001	1.50(0.46–4.86)	0.503
Chronic urinary disease	4.49(2.45–8.22)	<0.001	0.43(0.15–1.20)	0.108
Cerebrovascular disease	6.11(3.42–10.92)	<0.001	1.09(0.44–2.74)	0.850
Chronic gastroenterology and liver disease	1.48(0.59–3.68)	0.402		
Active malignancy	3.29(1.59–6.82)	0.001	5.94(1.69–20.87)	0.005
NEWS2 scores >2	139.51(56.22–346.21)	<0.001	124.88(37.14–419.87)	<0.001

COVID-19, Coronavirus disease 2019; OR, Odds ratios; NEWS2, National Early Warning Score 2.

### Clinical characteristics and independent predictors of false positive cases

There were 30 false positive cases and 86 true positive cases in this study. A significant difference was observed in age, the proportion of ≥65 years, hypertension, the numbers of comorbidities, SpO_2_, SBP, prehospital care (except for mechanical ventilation), NEWS2 scores, and PRIEST scores between false and true positive cases (*p* < 0.05) (Table 5). The patients who developed false positive were used as the dependent variable, and age ≥65 years, male, comorbidities, and prehospital parameters were used as the independent variables in the univariate logistic regression. Age ≥65 years, hypertension, diabetes, and SpO_2_ were screened out as meaningful variables and used as inputs for multivariate logistic regression (*p* < 0.1) (Table 6). Multivariate logistic regression showed that SpO_2_ (adjusted OR =1.87, *p* < 0.001) was the independent predictor for discriminating false positive patients from true positive patients (Table 6). Furthermore, the proportion of SPO_2_ >95% of false positive patients (70.0%) was higher than that of true positive patients (14.0%, *p* < 0.001).

**Table 5.** Clinical characteristics between false positive cases and true positive cases.

Variables	False positive (*n* = 30)	True positive (*n* = 86)	*P*
Age	83.0 (47.5–94.0)	89.5 (80.8–94.3)	0.047
≥65 years, n (%)	21 (70.0)	79 (91.9)	0.007
Male, n (%)	21 (70.0)	61 (70.9)	0.923
Comorbidities, n (%)			
Hypertension	10 (33.3)	64 (74.4)	<0.001
Diabetes	7 (23.3)	37 (43.0)	0.056
Chronic cardiac disease	16 (53.3)	53 (61.6)	0.426
Chronic pulmonary disease	5 (16.7)	14 (16.3)	1.000
Chronic urinary disease	9 (30.0)	26 (30.2)	0.981
Cerebrovascular disease	11 (36.7)	31 (36.0)	0.951
Chronic gastroenterology and liver disease	3 (10.0)	7 (8.1)	1.000
Active malignancy	2 (6.7)	12 (14.0)	0.466
Numbers of comorbidities	1.0 (0.0–2.0)	3.0 (2.0–4.0)	0.016
Prehospital physiological parameters			
SpO_2_ (%)	98.0 (95.0–99.0)	93.0 (91.0–94.0)	<0.001
Temperature (°C)	36.8 (36.5–38.0)	37.0 (36.5–37.6)	0.987
Respiratory rate (breaths/minute)	20.5 (18.0–24.3)	20.0 (18.0–21.0)	0.121
Pulse rate (beats/minute)	88.0 (72.0–99.8)	79.0 (72.0–93.0)	0.213
SBP (mmHg)	129.5 (121.0–137.3)	135.0 (124.0–150.5)	0.040
DBP (mmHg)	77.0 (71.5–84.0)	76.5 (71.0–85.3)	0.877
MAP (mmHg)	93.8 (88.6–100.3)	96.0 (89.6–104.3)	0.254
GCS	15.0 (10.0–15.0)	15.0 (13.0–15.0)	0.886
Prehospital care			
Oxygen therapy	15 (50.0)	86 (100.0)	<0.001
Transfusion	1 (3.3)	46 (53.5)	<0.001
ECG monitoring	5 (16.7)	64 (74.4)	<0.001
Mechanical ventilation	0 (0.0)	11 (12.8)	0.200
Use stretcher or wheelchair	19 (63.3)	76 (88.4)	0.002
Numbers of treatment measures	1.0 (0.0–2.0)	3.0 (2.0–4.0)	<0.001
Number of medical staff	2.0 (1.0–2.0)	2.0 (2.0–2.0)	0.001
Total transport time (minutes)	34.0 (9.8–42.5)	44.0 (42.0–46.0)	<0.001
Illness severity scores			
qSOFA	0.0 (0.0–1.0)	0.0 (0.0–1.0)	0.795
RAPS	2.0 (0.0–2.3)	1.0 (0.0–2.0)	0.385
REMS	7.0 (4.8–8.0)	7.0 (6.0–9.0)	0.222
CRB-65	1.0 (1.0–2.0)	1.0 (1.0–2.0)	0.081
NEWS2	4.0 (3.0–5.3)	6.0 (4.0–8.0)	0.001
PRIEST	12.5 (8.5–14.0)	13.5 (11.0–16.0)	0.003

SpO_2_, oxygen saturation; SBP, systolic blood pressure; DBP, diastolic blood pressure; MAP, mean arterial pressure; GCS, Glasgow coma scale; ECG, Electrocardiogram; qSOFA, quick Sequential Organ Failure Assessment; RAPS, Rapid Acute Physiology Score; REMS, Rapid Emergency Medicine Score; NEWS2, National Early Warning Score 2; PRIEST, Pandemic Respiratory Infection Emergency System Triage.

**Table 6.** Independent predictors for false positive cases.

Variables	Univariate logistic regression		Multivariate logistic regression	
	OR (95% CI)	*P*	Adjusted OR (95% CI)	*P*
Age ≥65 years	0.20(0.07–0.62)	0.005	0.96(0.17–5.30)	0.958
Male	0.96(0.39–2.37)	0.923		
Hypertension	0.17(0.07–0.42)	<0.001	0.36(0.11–1.22)	0.101
Diabetes	0.40(0.16–1.04)	0.060	0.54(0.15–1.98)	0.350
Chronic cardiac disease	0.71(0.31–1.65)	0.427		
Chronic pulmonary disease	1.03(0.34–3.15)	0.961		
Chronic urinary disease	0.99(0.40–2.45)	0.981		
Cerebrovascular disease	1.03(0.43–2.44)	0.951		
Chronic gastroenterology and liver disease	1.25(0.30–5.20)	0.755		
Active malignancy	0.44(0.09–2.09)	0.303		
SpO_2_ (%)	1.98(1.54–2.56)	<0.001	1.87(1.43–2.44)	<0.001
Temperature (°C)	1.18(0.73–1.91)	0.505		
Respiratory rate (breaths/minute)	1.08(0.98–1.19)	0.132		
Pulse rate (beats/minute)	1.01(0.99–1.04)	0.255		
SBP (mmHg)	0.98(0.97–1.00)	0.105		
DBP (mmHg)	0.99(0.95–1.03)	0.549		
MAP (mmHg)	0.98(0.94–1.01)	0.179		
GCS	0.95(0.82–1.11)	0.539		

OR, odds ratios; SpO_2_, oxygen saturation; SBP, systolic blood pressure; DBP, diastolic blood pressure; MAP, mean arterial pressure; GCS, Glasgow coma scale.

## Discussion

This study on the performance of prehospital physiological parameters related scores for discriminating the severe/critical state in patients with COVID-19 has three main findings. First, when >2 is selected as the threshold, prehospital NEWS2 scores as the best performer can accurately discriminate the severe/critical state in adult patients with COVID-19 and are an independent predictor for severity of illness categories of them. Second, the age, numbers of comorbidities, prehospital care workload, and consumption of medical human resources of patients with NEWS2 scores >2 are all higher than those of patients with NEWS2 scores ≤2. Third, SpO_2_ is the unique independent predictive variable influencing the discordance between false and true positive cases.

The pathogenesis of serious COVID-19 is related to immune response and host genetic background. There are severely dysfunctional adaptive immune response and hyperinflammatory innate immune system in patients with serious COVID-19. Their inflammatory markers are higher than those of patients in mild/moderate state [16, 17]. Genetic variations in SARS-CoV-2 receptor angiotensin-converting enzyme 2 or interferon signaling pathway may lead to the occurrence of serious COVID-19 [18]. Serious COVID-19 patients also show some clinical manifestations. Compared with patients in mild/moderate state, severe/critical patients are older, show rapid progression, and have more comorbidities, higher hospital admission rates, and higher ICU admission rates [19]. There are obvious differences in the antiviral treatment regimen and the timing of corticosteroid use between mild/moderate state and severe/critical state [20]. Serious patients may use invasive mechanical ventilation and extracorporeal membrane oxygenation during treatment [21]. Patients in severe/critical state also have higher mortality rates and lower health-related quality of life than those in mild/moderate state [6, 19]. In addition, some severe/critical patients exhibit silent hypoxemia, which makes it difficult to discriminate them from those in mild/moderate state through clinical manifestations at the initial stage [22]. Our study further showed that severe/critical patients have the clinical characteristics of older age, more numbers of comorbidities, lower SpO_2_, and poorer consciousness at the prehospital stage. Compared with mild/moderate patients, they have higher illness severity scores, greater prehospital care workload, and stronger consumption of medical human resources. Therefore, timely identifying the severity of COVID-19 at the prehospital stage is absolutely essential for reasonably arranging medical staff, predicting the prehospital care workload, and conducting triage for patients, especially in poor healthcare resource settings.

qSOFA, RAPS, REMS, CRB-65, NEWS2, and PRIEST were included in this study to assess the illness severity of patients with COVID-19 at the beginning of prehospital transport. qSOFA is a fast tool for screening suspected sepsis, and its performance in evaluating the prognosis of COVID-19 is mediocre [6, 9, 23]. CRB-65, as a modified version of CURB-65, is a clinical tool developed for risk stratification in patients with community-acquired pneumonia. It is convenient to use by clinicians without laboratory test for urea nitrogen, and it shows a good predictive value in evaluating the prognosis of hospitalized patients with critically ill COVID-19 [9, 24, 25]. Compared with qSOFA, CRB-65 can better identify patients with COVID-19 at risk for intensive respiratory or vasopressor support [26]. RAPS and REMS are commonly used to assess the transport risk and mortality of patients in emergency departments and hospital dispositions. REMS adds age and SpO_2_ on the basis of RAPS, and its overall predictive value for the prognosis of hospitalized patients with COVID-19 is higher than that of RAPS [8, 27–30]. It also shows a good predictive role for the outcomes of prehospital patients with COVID-19 [31]. NEWS2 is used for the identification of high-risk patients and clinical deterioration. It includes SpO_2_ and supplemental oxygen requirement, which are particularly important in assessing patients with COVID-19. The Royal College of Physicians (RCP) categorized the conditions of patients into four levels based on NEWS2 scores: low (0–4), low-medium (3 in any individual parameter), medium (5–6), and high (≥7) clinical risk [14]. With the increase of clinical risk, the in-hospital mortality of patients with COVID-19 increases [7]. NEWS2 is accurate and rapid in predicting the prognosis and clinical deterioration of hospitalized patients with COVID-19 when its threshold is ≥3–6 [6, 32–35], and its predictive value is better than those of qSOFA, CRB-65, and REMS [6, 36]. As a modification of NEWS2, PRIEST has a similar ability to predict the prognosis of patients with confirmed or suspected COVID-19 to NEWS2 [37, 38]. However, the application of these illness severity scores in the prehospital discrimination of severe/critical state in confirmed COVID-19 is still lacking in clinical practice. Compared with qSOFA, RAPS, REMS, and CRB-65, which are commonly used illness severity scores in prehospital and emergency, NEWS2 and PRIEST had better predictive performance for rapid identification of serious COVID-19 at the prehospital stage in our study. At the optimal cutoff value, the sensitivity and specificity of NEWS2 and PRIEST were 93.5%, 90.7% and 87.0%, 91.9%, respectively. NEWS2 not only had three fewer clinical parameters than PRIEST and was more convenient to use in a tense prehospital environment but also had better sensitivity and Youden index than PRIEST. Different from the threshold recommended by the RCP [14] and the thresholds for predicting the outcome of COVID-19 in other studies [6, 32–35], our study shows that the optimal threshold of NEWS2 is >2. This result may be related to the following factors. First of all, the threshold of NEWS2 recommended by the RCP is for the assessment and response of death and intensive care need in sepsis, not specifically for COVID-19 [14]. Secondly, the thresholds of other studies on COVID-19 are aimed at predicting the prognosis, while the threshold of this study is aimed at discriminating severe/critical COVID-19. This study and other studies have confirmed that the NEWS2 scores of non-survivor and deteriorated patients before and after admission are significantly higher than those of survivor and stable patients, respectively [6, 32–35]. Thirdly, the variants of SARS-CoV-2, the proportion of severe/critical patients, race, treatment methods and the definition of outcomes in different studies may be different [6, 7, 32–35]. Fourth, the condition of COVID-19 has dynamic changes, and some patients with lower prehospital NEWS2 may also deteriorate after admission, resulting in severe/critical COVID-19 or even death. Finally, the occurrence of serious COVID-19 is multifactorial. In addition to the NEWS2, age ≥65 years, hypertension and active malignancy are also independent predictors to discriminate severe/critical COVID-19 in this study. Even patients with lower prehospital NEWS2 may experience exacerbation if combined with other risk factors. Compared with predictive models and markers composed of laboratory results [4, 5, 39], NEWS2 can be obtained by measuring physiological parameters without blood or bronchoalveolar lavage test and can be used in risk stratification to predict serious patients quickly and cheaply. This study also suggested that NEWS2 has positive correlations with prehospital care workload and consumption of medical human resources, and it is an important independent predictor for severe/critical categories of COVID-19. When NEWS2 exceeds the threshold value, it is likely to indicate certain clinical significance. First, the prehospital care workload may expand, which requires the preparation of relevant medical equipment and adequate treatment measures to prevent possible equipment obstacles and adverse events in patients during transportation [40]. Second, more medical staff may need to participate in busy prehospital care and transport, and reasonable rest and psychosocial support can help them reduce psychological and social fear [41, 42]. Third, given that these patients are likely to suffer from serious COVID-19, multiple comorbidities, and advanced age, early recognition and timely triage to ICU or high-dependency unit are essential. This risk stratification based on NEWS2 may help clinicians make decisions fast, allowing them to pay considerable attention and medical resources to those identified as high risk for severe/critical state.

Our study found that distinguishing between false and true positive cases is necessary because the numbers of comorbidities, illness severity, prehospital care workload, and human resources consumption of true positive cases are higher than those of false positive cases. This study further demonstrated that SpO_2_ is the only independent predictor for identifying false and true positive cases. NEWS2 > 2 combined with a lower level of SpO_2_ than normal means a high likelihood of true positive case. Prehospital lowest SpO_2_ can also effectively predict the mortality of patients with COVID-19. A 1% decrease in prehospital SpO_2_ indicates a 13% increase in the odds of death [43]. Continuous monitoring of SpO_2_ and oxygen support has important clinical value for patients with serious COVID-19 [44]. Therefore, for patients with high prehospital NEWS2 scores and low SpO_2_, especially patients with silent hypoxemia, clinicians need to give them increased attention and carry out oxygen therapy or even mechanical ventilation during prehospital transport period.

This study has some limitations. First, the changes in vital signs of patients during the prehospital transport period were not fully recorded, making it difficult to assess the predictive effect of NEWS2 on the outcomes of prehospital treatment. Second, the transportation distance of each patient was not accurately recorded, so the absolute transportation time and workload were impossible to calculate. Third, this study is a single-center and retrospective study, and we hope to conduct additional prospective studies with multiple centers with larger sample size in the future.

## Conclusions

The prehospital NEWS2 and its modification PRIEST can effectively and rapidly discriminate serious COVID-19. High levels of NEWS2 suggest that prehospital care workload and the consumption of medical human resources will increase. For patients with NEWS2 > 2, lower SpO_2_ than normal level indicates the presence of true severe/critical state. Our findings have implications for the risk stratification and prehospital treatment of patients with COVID-19.

## Data Availability

The datasets used and/or analysed during the current study are available from the corresponding author upon reasonable request. If you need supporting data, you can contact us at any time. Email: tanjy302@163.com; suhaibin302@163.com.

## References

[1] Zhang L, Zhang Y, Duan W, et al. Using an influenza surveillance system to estimate the number of SARS-CoV-2 infections in Beijing, China, weeks 2 to 6 2023. Euro Surveil. 2023;28(11):1. doi: 10.2807/1560-7917.ES.2023.28.11.2300128. PMC10021470 36927716

[2] National Institutes of Health. Coronavirus Disease (COVID-19). 2019. Treatment guidelines. Clinical spectrum. https://www.covid19treatmentguidelines.nih.gov/. 34003615

[3] Chen N, Zhou M, Dong X, et al. Epidemiological and clinical characteristics of 99 cases of 2019 novel coronavirus pneumonia in Wuhan, China: a descriptive study. Lancet. 2020;395(10223):507–12. doi: 10.1016/S0140-6736(20)30211-7. 32007143 PMC7135076

[4] Velavan TP, Meyer CG. Mild versus severe COVID-19: laboratory markers. Int J Infect Dis. 2020;95:304–307. doi: 10.1016/j.ijid.2020.04.061. 32344011 PMC7194601

[5] Bao S, Lu G, Kang Y, et al. A diagnostic model for serious COVID-19 infection among older adults in Shanghai during the omicron wave. Front Med (Lausanne). 2022;9:1018516. doi: 10.3389/fmed.2022.1018516. 36600892 PMC9806114

[6] Liu FY, Sun XL, Zhang Y, et al. Evaluation of the risk prediction tools for patients with coronavirus disease 2019 in Wuhan, China: a single-centered, retrospective, observational study. Crit Care Med. 2020;48(11):e1004-11–e1011. doi: 10.1097/CCM.0000000000004549. 32897668 PMC7448719

[7] Rigoni M, Torri E, Nollo G, et al. NEWS2 is a valuable tool for appropriate clinical management of COVID-19 patients. Eur J Intern Med. 2021;85:118–120. doi: 10.1016/j.ejim.2020.11.020. 33358535 PMC7751376

[8] Vedovati MC, Barbieri G, Urbini C, et al. Clinical prediction models in hospitalized patients with COVID-19: a multicenter cohort study. Respir Med. 2022;202:106954. doi: 10.1016/j.rmed.2022.106954. 36057141 PMC9392655

[9] Fan G, Tu C, Zhou F, et al. Comparison of severity scores for COVID-19 patients with pneumonia: a retrospective study. Eur Respir J. 2020;56(3):2002113. doi: 10.1183/13993003.02113-2020. 32675205 PMC7366179

[10] Singer M, Deutschman CS, Seymour CW, et al. The third international consensus definitions for sepsis and septic shock (sepsis-3). JAMA. 2016;315(8):801–810. doi: 10.1001/jama.2016.0287. 26903338 PMC4968574

[11] Rhee KJ, Fisher CJ, Jr, Willitis NH. The rapid acute physiology score. Am J Emerg Med. 1987;5(4):278–282. doi: 10.1016/0735-6757(87)90350-0. 3593492

[12] Olsson T, Lind L. Comparison of the rapid emergency medicine score and APACHE II in nonsurgical emergency department patients. Acad Emerg Med. 2003;10(10):1040–1048. doi: 10.1197/S1069-6563(03)00342-7. 14525735

[13] Lim WS, van der Eerden MM, Laing R, et al. Defining community acquired pneumonia severity on presentation to hospital: an international derivation and validation study. Thorax. 2003;58(5):377–382. doi: 10.1136/thorax.58.5.377. 12728155 PMC1746657

[14] Royal College of Physicians. 2017. National Early Warning Score (NEWS) 2. https://www.rcplondon.ac.uk/projects/outputs/national-early-warning-score-news-2

[15] Goodacre S, Thomas B, Sutton L, et al. Derivation and validation of a clinical severity score for acutely ill adults with suspected COVID-19: the PRIEST observational cohort study. PLoS One. 2021;16(1):e0245840. doi: 10.1371/journal.pone.0245840. 33481930 PMC7822515

[16] Janssen NAF, Grondman I, de Nooijer AH, et al. Dysregulated innate and adaptive immune responses discriminate disease severity in COVID-19. J Infect Dis. 2021;223(8):1322–1333. doi: 10.1093/infdis/jiab065. 33524124 PMC7928798

[17] Maamari KA, Busaidi IA, Kindi MA, et al. Short and long-term immune changes in different severity groups of COVID-19 disease. Int J Infect Dis. 2022;122:776–784. doi: 10.1016/j.ijid.2022.07.026. 35840099 PMC9284586

[18] Brest P, Mograbi B, Gal J, et al. Host genetic variability and determinants of severe COVID-19. Trends Genet. 2023;39(3):169–171. doi: 10.1016/j.tig.2022.10.003. 36379742 PMC9652763

[19] Verveen A, Wynberg E, van Willigen HDG, et al. Health-related quality of life among persons with initial mild, moderate, and severe or critical COVID-19 at 1 and 12 months after infection: a prospective cohort study. BMC Med. 2022;20(1):422. doi: 10.1186/s12916-022-02615-7. 36324167 PMC9629769

[20] Update to living WHO guideline on drugs for COVID-19. BMJ. 2023;380:p57. doi: 10.1136/bmj.p57. 36634963

[21] Cheng L, Bai WH, Yang JJ, et al. Construction and validation of mortality risk nomograph model for severe/critical patients with COVID-19. Diagnostics (Basel). 2022;12(10):2562. doi: 10.3390/diagnostics12102562. 36292251 PMC9601583

[22] Le Borgne P, Oberlin M, Bassand A, et al. Pre-hospital management of critically ill patients with SARS-CoV-2 infection: a retrospective multicenter study. J Clin Med. 2020;9(11):3744. doi: 10.3390/jcm9113744. 33233324 PMC7700636

[23] Khari S, Salimi Akin Abadi A, Pazokian M, et al. CURB-65, qSOFA, and SIRS criteria in predicting in-hospital ­mortality of critically ill COVID-19 patients: a prognostic accuracy study. Arch Acad Emerg Med. 2022;10(1):e36. 35765619 10.22037/aaem.v10i1.1565 PMC9187131

[24] Bellos I, Lourida P, Argyraki A, et al. Development of a novel risk score for the prediction of critical illness amongst COVID-19 patients. Int J Clin Pract. 2021;75(4):e13915. doi: 10.1111/ijcp.13915. 33969593 PMC7883033

[25] Tyagi A, Tyagi S, Agrawal A, et al. Early warning scores at time of ICU admission to predict mortality in critically ill COVID-19 patients. Disaster Med Public Health Prep. 2021. doi: 10.1017/dmp.2021.208. PMC8376854 34140066

[26] Su Y, Tu GW, Ju MJ, et al. Comparison of CRB-65 and quick sepsis-related organ failure assessment for predicting the need for intensive respiratory or vasopressor support in patients with COVID-19. J Infect. 2020;81(4):647–679. doi: 10.1016/j.jinf.2020.05.007. PMC7204730 32389785

[27] Covino M, Sandroni C, Santoro M, et al. Predicting intensive care unit admission and death for COVID-19 patients in the emergency department using early warning scores. Resuscitation. 2020;156:84–91. doi: 10.1016/j.resuscitation.2020.08.124. 32918985 PMC7480278

[28] Hu H, Yao N, Qiu Y. Comparing rapid scoring systems in mortality prediction of critically ill patients with novel coronavirus disease. Acad Emerg Med. 2020;27(6):461–468. doi: 10.1111/acem.13992. 32311790 PMC7264631

[29] Khari S, Zandi M, Zarmehrparirouy M, et al. Prognostic value of physiological scoring systems in COVID-19 patients: a prospective observational study. Adv Emerg Nurs J. 2023;45(1):77–85. doi: 10.1097/TME.0000000000000445. 36757751

[30] Hu H, Kong W, Yao N, et al. Prognostic value of three rapid scoring scales and combined predictors for the assessment of patients with coronavirus disease 2019. Nurs Open. 2022;9(3):1865–1872. doi: 10.1002/nop2.934. 34080790 PMC8242648

[31] Bourn SS, Crowe RP, Fernandez AR, et al. Initial prehospital rapid emergency medicine score (REMS) to predict outcomes for COVID-19 patients. J Am Coll Emerg Physicians Open. 2021;2(4):e12483. 34223444 10.1002/emp2.12483 PMC8240529

[32] Kostakis I, Smith GB, Prytherch D, Portsmouth Academic Consortium For Investigating COVID-19 (PACIFIC-19)., et al. The performance of the national early warning score and national early warning score 2 in hospitalised patients infected by the severe acute respiratory syndrome coronavirus 2 (SARS-CoV-2). Resuscitation. 2021;159:150–157. doi: 10.1016/j.resuscitation.2020.10.039. 33176170 PMC7648887

[33] Su Y, Ju MJ, Xie RC, et al. Prognostic accuracy of early warning scores for clinical deterioration in patients with COVID-19. Front Med (Lausanne). 2021;7:624255. doi: 10.3389/fmed.2020.624255. 33598468 PMC7882600

[34] Luo Z, Peng X, Zhou F, et al. Using NEWS2 to triage newly admitted patients with COVID-19. Nurs Crit Care. 2023;28(3):388–395. doi: 10.1111/nicc.12739. 34889010

[35] De Socio GV, Gidari A, Sicari F, et al. National early warning score 2 (NEWS2) better predicts critical coronavirus disease 2019 (COVID-19) illness than COVID-GRAM, a multi-centre study. Infection. 2021;49(5):1033–1038. doi: 10.1007/s15010-021-01620-x. 33970431 PMC8108728

[36] Myrstad M, Ihle-Hansen H, Tveita AA, et al. National early warning score 2 (NEWS2) on admission predicts severe disease and in-hospital mortality from COVID-19 – a prospective cohort study. Scand J Trauma Resusc Emerg Med. 2020;28(1):66. doi: 10.1186/s13049-020-00764-3. 32660623 PMC7356106

[37] Marincowitz C, Sutton L, Stone T, et al. Prognostic accuracy of triage tools for adults with suspected COVID-19 in a prehospital setting: an observational cohort study. Emerg Med J. 2022;39(4):317–324. doi: 10.1136/emermed-2021-211934. 35140074 PMC8844966

[38] Heydari F, Zamani M, Masoumi B, et al. Physiologic scoring systems in predicting the COVID-19 patients’ one-month mortality: a prognostic accuracy study. Arch Acad Emerg Med. 2022;10(1):e83. 36426162 10.22037/aaem.v10i1.1728 PMC9676706

[39] Zhang Y, Han J, Sun F, et al. A practical scoring model to predict the occurrence of critical illness in hospitalized patients with SARS-CoV-2 omicron infection. Front Microbiol. 2022;13:1031231. doi: 10.3389/fmicb.2022.1031231. 36601398 PMC9806124

[40] Baru A, Sultan M, Beza L. The status of prehospital care delivery for COVID-19 patients in Addis Ababa, Ethiopia: the study emphasizing adverse events occurring in prehospital transport and associated factors. PLoS One. 2022;17(2):e0263278. doi: 10.1371/journal.pone.0263278. 35104287 PMC8806066

[41] Sahin CE, Deger MS, Sezerol MA, et al. Covid-19 phobia in prehospital emergency medical services workers in Turkey. Niger J Clin Pract. 2022;25(8):1239–1246. doi: 10.4103/njcp.njcp_2035_21. 35975370

[42] Chang YT, Hu YJ. Burnout and health issues among prehospital personnel in Taiwan fire departments during a sudden spike in community COVID-19 cases: a cross-sectional study. Int J Environ Res Public Health. 2022;19(4):2257. doi: 10.3390/ijerph19042257. 35206444 PMC8872158

[43] Dillon K, Hook C, Coupland Z, et al. Pre-hospital lowest recorded oxygen saturation independently predicts death in patients with COVID-19. Br Paramed J. 2020;5(3):59–65. doi: 10.29045/14784726.2020.09.5.3.59. 33456398 PMC7783956

[44] Sobel JA, Levy J, Almog R, et al. Descriptive characteristics of continuous oximetry measurement in moderate to severe COVID-19 patients. Sci Rep. 2023;13(1):442. doi: 10.1038/s41598-022-27342-0. 36624254 PMC9828367

